# Differential White Blood Cell Count and Type 2 Diabetes: Systematic Review and Meta-Analysis of Cross-Sectional and Prospective Studies

**DOI:** 10.1371/journal.pone.0013405

**Published:** 2010-10-18

**Authors:** Effrossyni Gkrania-Klotsas, Zheng Ye, Andrew J. Cooper, Stephen J. Sharp, Robert Luben, Mary L. Biggs, Liang-Kung Chen, Kuppan Gokulakrishnan, Markolf Hanefeld, Erik Ingelsson, Wen-An Lai, Shih-Yi Lin, Lars Lind, Vitool Lohsoonthorn, Viswanathan Mohan, Antonio Muscari, Goran Nilsson, John Ohrvik, Jiang Chao Qiang, Nancy Swords Jenny, Koji Tamakoshi, Theodora Temelkova-Kurktschiev, Ya-Yu Wang, Chittaranjan Sakerlal Yajnik, Marco Zoli, Kay-Tee Khaw, Nita G. Forouhi, Nicholas J. Wareham, Claudia Langenberg

**Affiliations:** 1 MRC Epidemiology Unit, Institute of Metabolic Science, Cambridge, United Kingdom; 2 Department of Public Health and Primary Care, University of Cambridge, Cambridge, United Kingdom; 3 Department of Biostatistics, School of Public Health, University of Washington, Seattle, Washington, United States of America; 4 Department of Family Medicine, Taipei Veterans General Hospital, National Yang Ming University School of Medicine, Taipei, Taiwan; 5 Madras Diabetes Research Foundation and Dr. Mohan's Diabetes Specialities Centre, Chennai, India; 6 Centre for Clinical Study, Gesellschaft für Wissens- und Technologietransfer, Technisches Universität Dresden GmBH, Dresden, Germany; 7 Department of Medical Epidemiology and Biostatistics, Karolinska Institutet, Stockholm, Sweden; 8 Department of Family Medicine, Kuang Tien General Hospital, Taichung City, Taiwan; 9 Division of Endrocrinology and Metabolism, Taichung Veterans General Hospital, Taichung City, Taiwan; 10 Department of Medical Sciences, Uppsala University Hospital, Uppsala, Sweden; 11 Department of Preventive and Social Medicine, Faculty of Medicine, Chulalongkorn University, Bangkok, Thailand; 12 Department of Internal Medicine, Aging and Nephrological Diseases, University of Bologna, Bologna, Italy; 13 Centre for Clinical Research, Uppsala University, Vasteras, Sweden; 14 Department of Medicine, Karolinska Institutet, Stockholm, Sweden; 15 Guangzhou No. 12 Hospital, Guangzhou, China; 16 Department of Pathology, College of Medicine, University of Vermont, Burlington, Vermont, United States of America; 17 Department of Nursing, Nagoya University School of Health Sciences, Nagoya, Japan; 18 Medicobiological Unit, International Scientific Institute, National Sports Academy and “Robert Koch” German Medical Center, Sofia, Bulgaria; 19 King Edward Memorial Hospital, Pune, India; University of Padova, Italy

## Abstract

**Objective:**

Biological evidence suggests that inflammation might induce type 2 diabetes (T2D), and epidemiological studies have shown an association between higher white blood cell count (WBC) and T2D. However, the association has not been systematically investigated.

**Research Design and Methods:**

Studies were identified through computer-based and manual searches. Previously unreported studies were sought through correspondence. 20 studies were identified (8,647 T2D cases and 85,040 non-cases). Estimates of the association of WBC with T2D were combined using random effects meta-analysis; sources of heterogeneity as well as presence of publication bias were explored.

**Results:**

The combined relative risk (RR) comparing the top to bottom tertile of the WBC count was 1.61 (95% CI: 1.45; 1.79, p = 1.5*10^−18^). Substantial heterogeneity was present (I^2^ = 83%). For granulocytes the RR was 1.38 (95% CI: 1.17; 1.64, p = 1.5*10^−4^), for lymphocytes 1.26 (95% CI: 1.02; 1.56, p = 0.029), and for monocytes 0.93 (95% CI: 0.68; 1.28, p = 0.67) comparing top to bottom tertile. In cross-sectional studies, RR was 1.74 (95% CI: 1.49; 2.02, p = 7.7*10^−13^), while in cohort studies it was 1.48 (95% CI: 1.22; 1.79, p = 7.7*10^−5^). We assessed the impact of confounding in EPIC-Norfolk study and found that the age and sex adjusted HR of 2.19 (95% CI: 1.74; 2.75) was attenuated to 1.82 (95% CI: 1.45; 2.29) after further accounting for smoking, T2D family history, physical activity, education, BMI and waist circumference.

**Conclusions:**

A raised WBC is associated with higher risk of T2D. The presence of publication bias and failure to control for all potential confounders in all studies means the observed association is likely an overestimate.

## Introduction

Chronic inflammation, characterized by the increased production of cytokines and acute-phase reactants and activation of inflammatory signalling networks [1]–[5], may be involved in the pathogenesis of type 2 diabetes (T2D).Various markers of inflammation have been shown to predict the future diabetes risk, including Interleukin-6 (IL-6) and C-reactive protein (CRP) [1], [5].Obesity, a strong risk factor for T2D is also associated with inflammation as fat tissue releases inflammatory cytokines[6], [7]. Inflammation on its own can affect insulin signalling [3], indirectly increasing the risk of T2D, without the presence of obesity. Inflammation is also thought to promote beta-cell death [8]. However, there is considerable uncertainty about the direction of causality of the relationship between inflammation and T2D.

Evidence from epidemiological studies suggests an association between total peripheral white blood cell (WBC) or leukocyte count, a non-specific marker of inflammation, and diabetes risk[9], [10]. Although a number of studies have been published, they have not been systematically reviewed or meta-analysed. Granulocytes themselves are comprised of neutrophils, basophils and eosinophils[9]. Little is known about the association of each of the subfractions with T2D.

In the present study we systematically review and meta-analyse existing studies of the association between differential WBC count and T2D, including previously unpublished data from 5,021 cases and 43,508 non-cases (with 499 cases and 15,051 non-cases from EPIC-Norfolk study) obtained through correspondence with investigators. We also explore the potential roles of reverse causality, publication bias and confounding.

## Methods

### A. Systematic review and meta-analysis

#### Bibliographic search, literature review and data extraction

**Figure 1:**
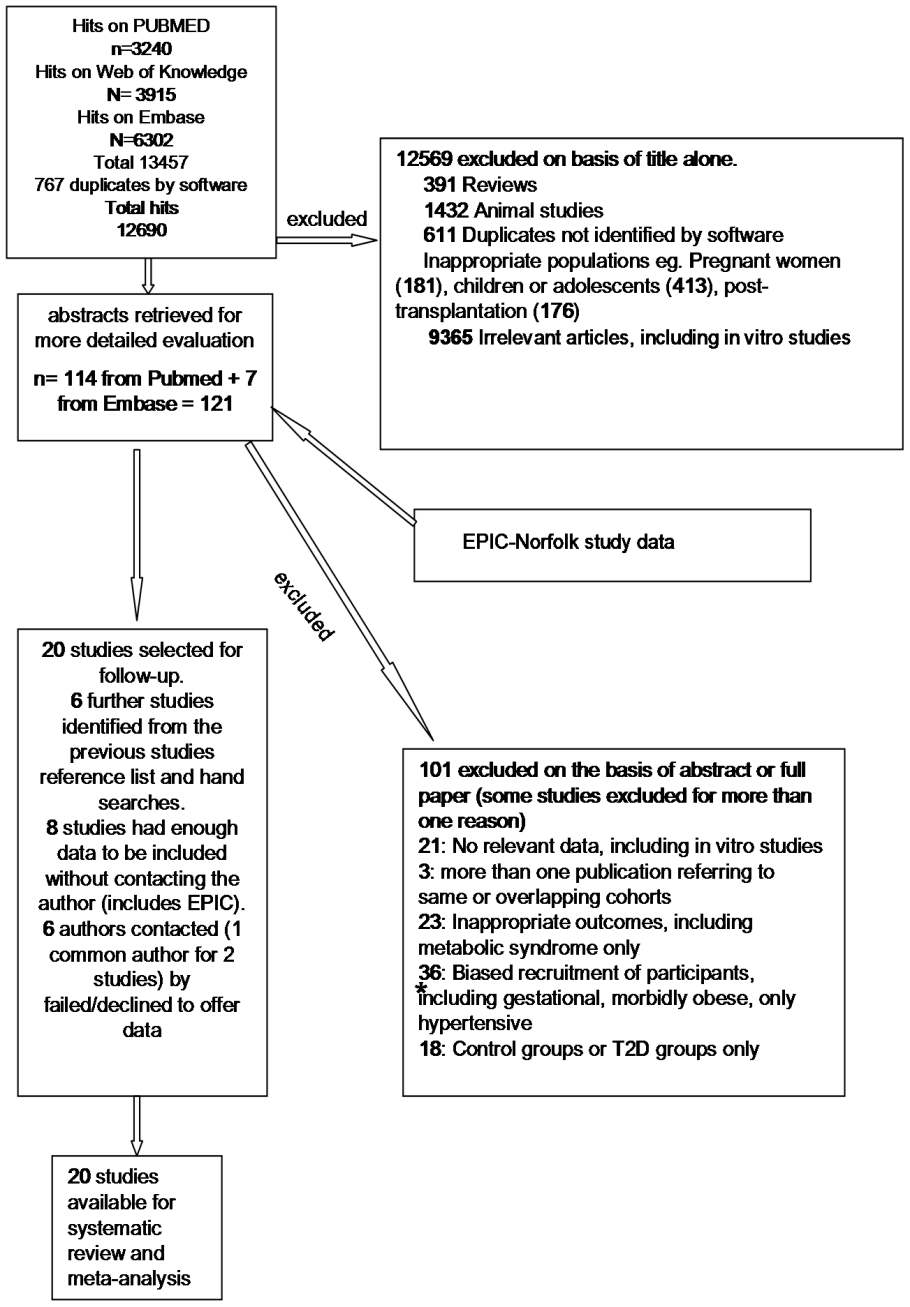
Information Flow Diagram.

A bibliographic search was conducted by the first author to identify all published evidence on the association between WBC or leukocyte (from now and on, WBC) count and T2D. The search terms included (“leukocyte” OR “leucocyte “OR “white blood”) combined (AND) with diabetes (diabetes” OR “glucose” OR “metabolic syndrome” OR “hyperglycaemia” OR “hyperglycemia”). We searched Pubmed 2.0 (National Library of Medicine) entering each search term as a MeSH, ISI Web of Knowledge^SM^ version 4.7 (©Thomson Reuters 2009) and Embase (© 2009 Elsevier B.V.), initially without limits with regard to publication date or language. Last searches were conducted in April 2010. Two authors (EGK, ZY) independently reviewed all identified titles (n = 12,705), and subsequently abstracts (n = 136) and full articles (Figure 1). We included evidence from cross-sectional and prospective cohort studies of adults that used standard definitions of T2D [11], adjusted for at least age, sex and BMI (excluded studies n = 1). No case-control studies were identified. For results from the same cohort published more than once (n = 3), we included the study with the largest sample reported (n = 1). We excluded studies of children and adolescents or with participants who had undergone solid organ or bone marrow transplantation (Figure 1). Discrepancies in articles selected for inclusion were addressed by consensus (n = 1). We additionally hand searched reference lists of all articles selected for inclusion. Two authors (EGK, ZY) extracted information from each article selected for inclusion including the number of cases and non-cases, study design and population, measurement of WBC and diagnosis of T2D, effect estimate and 95% confidence intervals for associations between WBC, neutrophil/granulocyte, lymphocyte and monocyte count and T2D risk. Where the risk ratio (odds ratio, relative risk or hazard ratio) was presented using other than three groups of WBC, these were converted to compare the top to bottom tertile of WBC [12].

We identified studies that appeared during the literature review and did not report on the association between WBC and T2D but had potentially collected pertinent data, as evident from their study description. Corresponding authors of these studies (n = 19) were contacted by electronic and regular mail (two electronic reminders) and invited to submit data using a standardized data extraction sheet and uniform analysis plan. We requested odds ratios, relative risks or hazard ratios for the association between WBC and its sub-fractions where available, comparing top to bottom tertile of each measure, adjusting for age, sex, smoking, BMI and waist circumference and using the WHO definition of diabetes[11]. Data on the number of cases and non-cases and 95% confidence intervals for the estimated effects were also requested.

We additionally included unpublished results from the EPIC Norfolk study, described in more detail below, according to the protocol used for obtaining unpublished evidence from other investigators.

#### Meta-analyses

For the purposes of the meta-analysis, we considered all of odds ratios, risk ratios and hazard ratios as estimates of the relative risk. These relative risks were combined across studies using random effects meta-analysis. Heterogeneity was assessed using the I^2^ statistic, which represents the proportion of variation in the effect sizes that is attributable to genuine differences across studies rather than to random error [13]. To identify potential sources of heterogeneity between studies and to assess the effect of study characteristics on the results, we repeated the meta-analysis in strata defined by study size, design and method of data collection and ethnicity of participants. The between-study variance was used to quantify the degree of heterogeneity among studies [14]. We also used meta-regression to estimate the effect of each of the covariates on the relative risk. Publication bias was assessed using a funnel plot and Begg and Egger tests [15], [16].Statistical analyses were performed using Stata (version 10.0) statistical software (Stata Corporation, College Station, Texas, USA). Results were presented in forest plots, where the sizes of the boxes for individual studies are inversely proportional to the variances of the log relative risks, and the horizontal lines represent 95% CIs.

### B. EPIC-Norfolk cohort

In addition to including results from the European Prospective Investigation of Cancer in Norfolk, UK (EPIC-Norfolk) as part of the overall meta-analysis according to the standard protocol described above, we used prospective data from this cohort to investigate the effect of adjusting for a range of potential confounding factors not consistently available across other studies.

#### EPIC-Norfolk participants

EPIC-Norfolk is a population-based cohort study, which has previously been described in detail [17]. In brief, men and women aged 40 to 79 years were eligible for participation. In total, 77,630 invitations were sent, 30,447 (39%) individuals consented to take part, with 25,639 (33%) attending the baseline health check between 1993 and 1997. The study was approved by the Norfolk Local Research Ethics Committee and all participants gave written informed consent.

We excluded participants with a history of stroke, myocardial infarction, or cancer (n = 2,460) or prevalent or unconfirmed diabetes at baseline (n = 688).

#### Measurements

A detailed self-completed health and lifestyle questionnaire was completed at baseline, including questions on family history of diabetes, prescribed medications, occupational social class, smoking status, educational level, and physical activity assessed by a four point index[17]. Participants were invited to attend a baseline health check-up at the study clinic where health checks were carried out by trained research nurses. Anthropometric measurements were taken according to standard protocol[17]. Two further health check-ups were performed, after an average follow-up time of three and thirteen years respectively. Since the baseline health-check visit, there were three follow-up assessments: a postal questionnaire at 18 months, a second health-check visit (1998–2000), and a further postal questionnaire (2002–2004).

#### Biochemical analyses

Biochemical assays were carried out on samples drawn with participants in the non-fasted state. Blood samples for WBC measurement were stored overnight at room temperature and were collected each morning and transported to the EPIC-Norfolk laboratory in Attleborough (UK). The samples were analysed in a random order using impedance counting technique with an MD18 haematology analyser (Coulter Corporation, Miami, FL, USA). Quality controls were carried out daily. In addition, the Haematology Department of Addenbrooke's Hospital included the EPIC Laboratory in a monthly quality control scheme. The WBC count coefficient of variation for the period of study was ≤3.0%. The standard deviation values for the differential granulocyte, lymphocyte and monocyte percentages were less than or equal to 1.5, 1.5, and 3.0, respectively [18]. Researchers and laboratory personnel did not have access to identifiable information, and could only identify samples by number.

#### Incident type 2 diabetes

Ascertainment of incident cases of type 2 diabetes involved review of multiple sources of evidence including self-report (self reported doctor diagnosed diabetes, anti-diabetic drug use) during follow-up, linkage to primary and secondary care registers, hospital admissions and mortality data[17]. Criteria for qualification as a confirmed diabetes case were: confirmation of self-report by another data source or diagnosis captured by an external source alone, independently of participation in study follow-up questionnaires or visit. Possible cases based solely on self-report and not confirmed by another data source did not qualify as a confirmed case of diabetes. Cases not meeting the above criteria were excluded (n = 5).

#### Statistical analysis

Cox proportional hazards regression was used to estimate hazard ratios for the incidence of diabetes by tertiles of WBC, granulocyte, lymphocyte, and monocyte count, using the lowest tertile as the reference category. Results from age and sex adjusted models were compared to those additionally adjusting for smoking status (never, former or current), waist circumference (continuous), BMI (continuous), educational level (below ‘A’ level vs. ‘A’ level and above where ‘A’ level is school education to age 18 years), a positive family history of diabetes, and physical activity level (4 categories ranging from sedentary to active).

Analyses were restricted to participants with full information on total WBC, granulocyte, lymphocyte or monocyte count (n = 15,708). We further excluded 158 participants without complete information on covariates or exclusion criteria.

15,550 participants (499 incident diabetes cases) remained in the analyses. We calculated a Health Behaviours Score (HBS), including information on physical activity, alcohol intake, plasma vitamin C (a biomarker of fruit and vegetable intake) and smoking, as proposed by Khaw et al [19] and compared models with and without an interaction term for HBS, using a likelihood ratio test.

All statistical analyses were performed using Stata/SE 10.0 (Stata-Corp, College Station, Texas, USA). All *p* values were based on 2-sided tests.

## Results

### A. Systematic review and meta-analysis

Initial searches identified 12,705 articles and abstracts (Figure 1). After exclusions, a total of 27 publications were included, 7 of which reported results on the association between WBC counts and T2D diabetes risk in a format that could be used[9], [10], [20]–[24]. We contacted 19 corresponding authors of the other 20 studies [25]–[44] and received data for 13[25], [27]–[29], [33], [34], [36], [37], [39], [40], [42]–[44]. A total of 6 authors (7 studies) did not respond (n = 6) or declined participation (n = 1). Tabular data from one study could not be used because it was not available in full [27].Our meta-analysis was therefore based on data from 20 independent studies (see Figure 1, Table S1 and PRISMA checklist S1), including EPIC-Norfolk results.

#### Combined results

**Figure 2:**
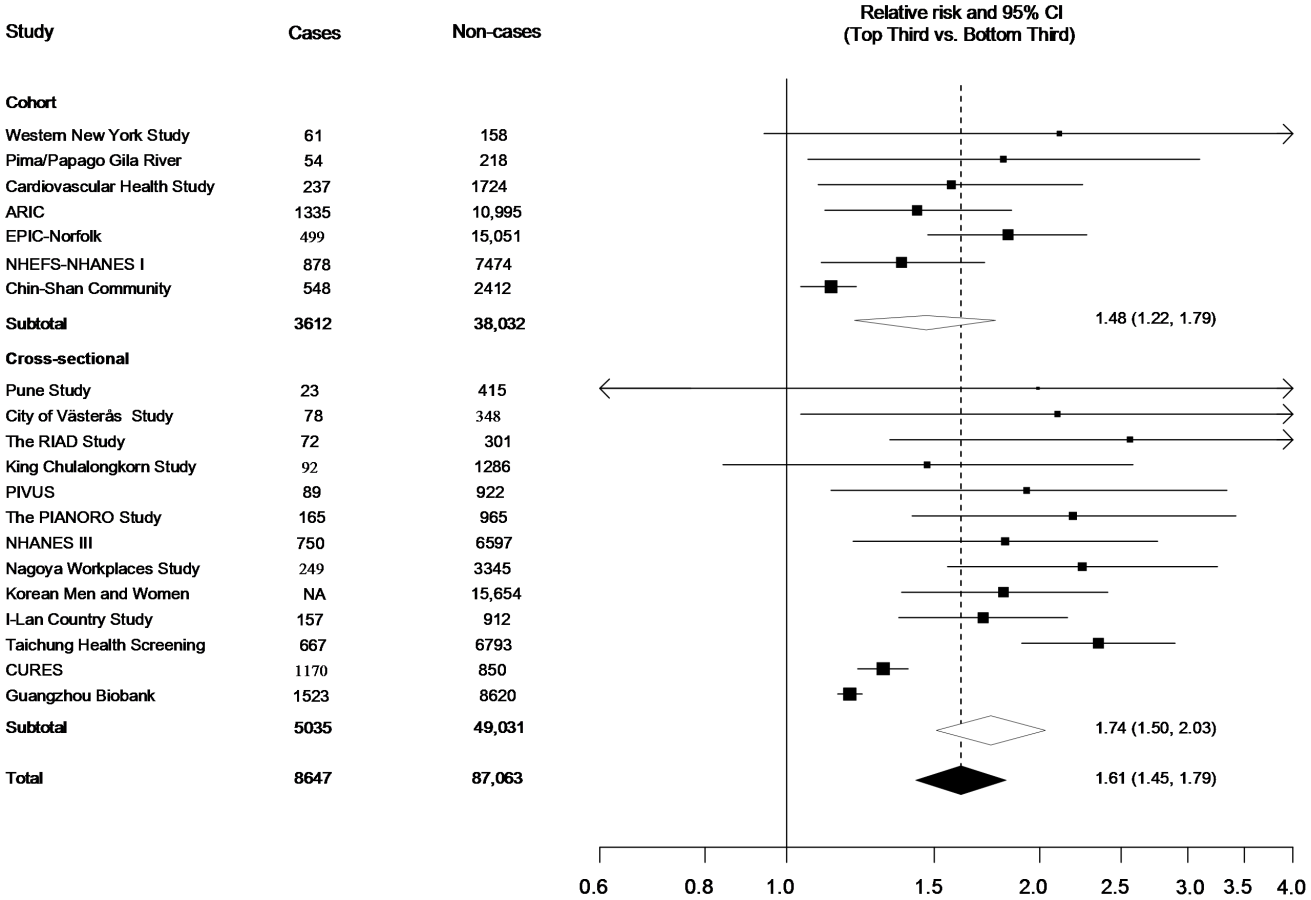
Forest plot showing study-specific and combined effect estimates comparing the top to bottom tertile of the WBC count distribution.

**Figure 3:**
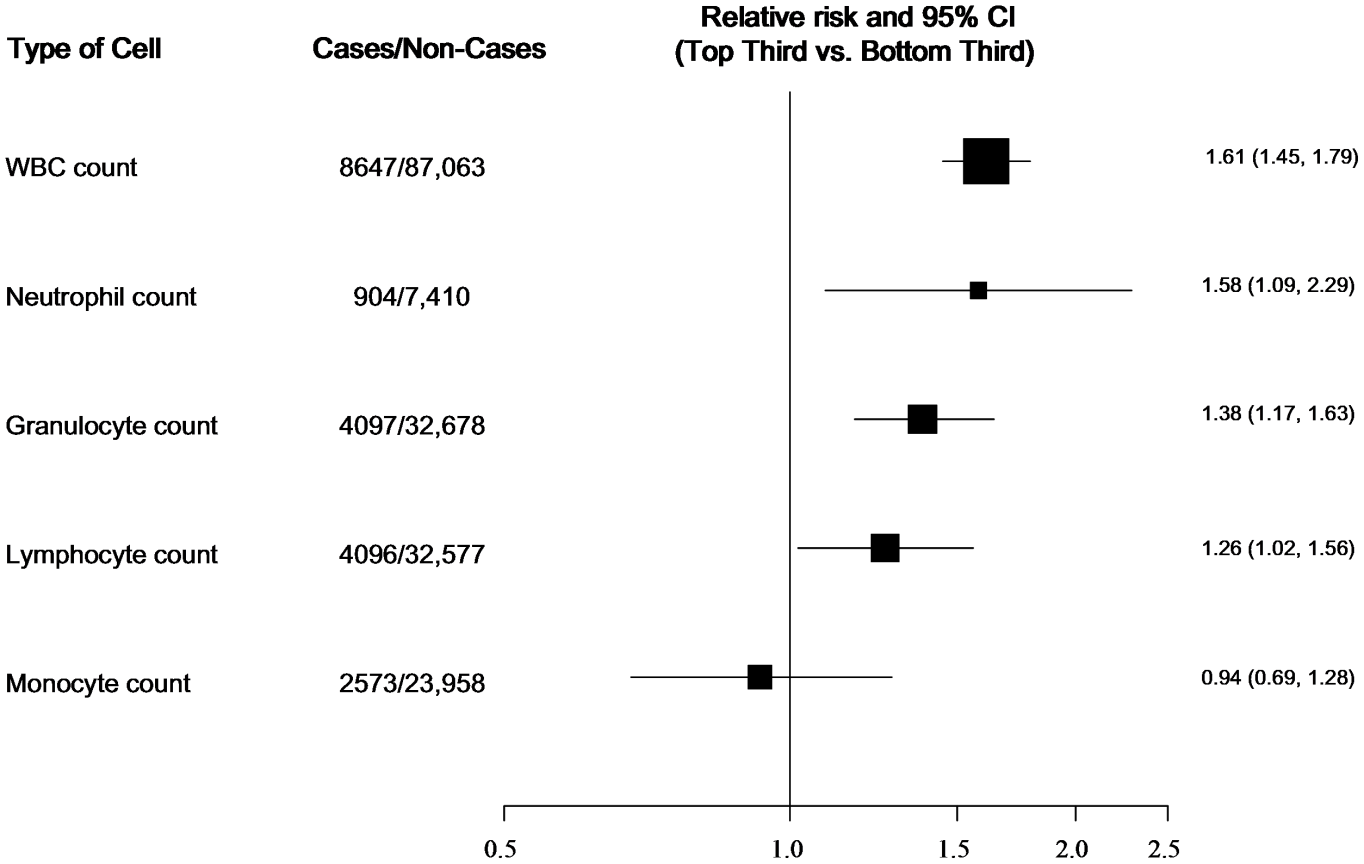
Forest plot showing combined effect estimates for T2D comparing the top to bottom tertile of the distribution of WBC sub-fractions (*Granulocytes include Neutrophils plus Eosinophils plus Basophils*).

The combined relative risk (RR) comparing the top to bottom tertile of the total WBC count distribution was 1.61 (95% CI: 1.45; 1.79, p = 1.5*10^−18^). (Figure 2). The combined relative risk was 1.38 (1.17; 1.64, p = 1.5*10^−4^) for granulocytes, 1.26 (1.02; 1.56, p = 0.029) for lymphocytes, and 0.93 (0.68; 1.28, p = 0.67) for monocytes, comparing the top to bottom tertile of the distribution of each measure (Figure 3).

#### Heterogeneity

**Figure 4:**
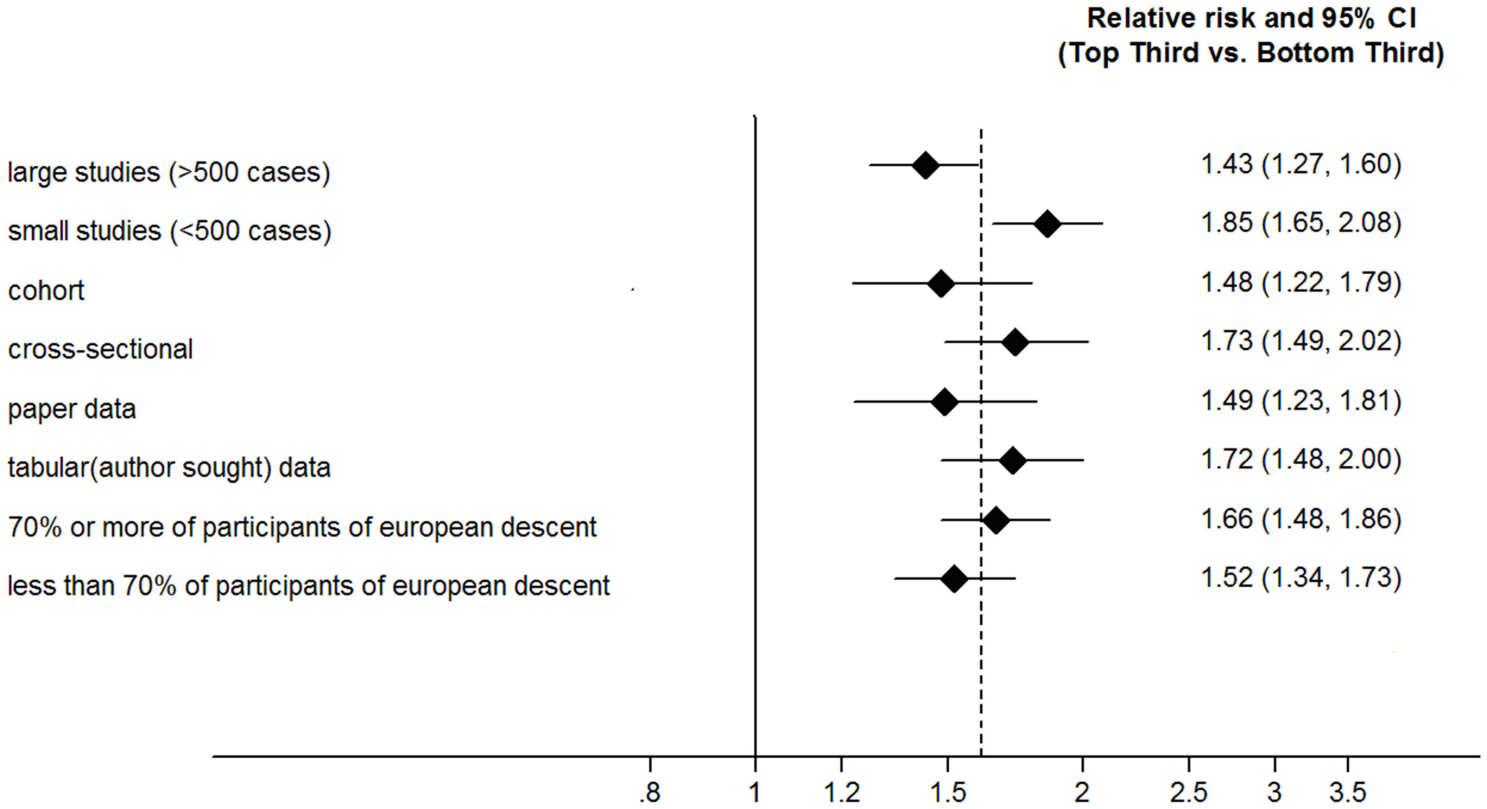
Forest plot showing combined effect estimates for T2D comparing the top to bottom tertile of the WBC count distribution. * *dotted line representing combined effect estimate for meta-analysis. Size of rhomboids not informative of weight*.

For total WBC, there was a high degree of heterogeneity between the 20 studies (I^2^ = 83%, p<0.001). I^2^ was slightly smaller but still statistically significant among prospective cohort studies (I^2^ = 74%, p<0.001). Sensitivity analyses were used to identify potential sources of heterogeneity between studies. Stratification by study design showed a combined RR for WBC count of 1.73 (95% CI: 1.49; 2.02, p = 7.7*10^−13^) for prevalent (5,035 cases; 47,008 non-cases) and 1.48 (95% CI: 1.22; 1.79, p = 7.7*10^−5^).for incident (3,612 cases; 38,032 non-cases) T2D. Subgroup analyses (Figure 4) revealed a lower RR in larger (≥500 cases) compared to smaller studies (RR 1.43, 95% CI: 1.27; 1.60,p = 3.4*10e-9) versus RR 1.85, 95% CI: 1.64; 2.08, p = 3.6*10^−25^). and no significant difference comparing published to unpublished evidence included in this report (RR 1.49, 95% CI: 1.23; 1.81, p = 5.9*10^−5^) versus RR 1.72, 95% CI: 1.48; 2.00, p = 1.1*10^−12^). The combined RR was 1.66 (95% CI 1.48–1.86, p = 2.8*10^−18^) versus 1.52 (95% CI 1.34–1.73, p = 2.02*10^−10^) when comparing studies including more than 70% versus less than 70% European descent participants respectively.

Including ethnicity (percentage of participants of European descent), number of cases, number of participants, source of data (investigator provided versus published data), and type of study (prospective cohort versus cross-sectional study or cross sectional data from a cohort study) in a meta-regression model resulted in a decrease in the value of I^2^ from 83% to 36.0%. The beta-coefficients and corresponding p values from the meta-regression models using each of the above parameters in turn are presented in table 1.

**Table 1:** β coefficients and corresponding p values from the meta-regression models.

Covariate	β coefficient	P value	N of studies
Source of data (tabular vs published paper)	0.144	0.28	20
Type of study (cross-sectional vs longitudinal)	0.164	0.213	20
Number of cases	−0.0003	0.018	19*
Number of participants	−0.000002	0.89	20
Percentage of Caucasian participants	0.142	0.316	20

*Number of cases not available for one study [*60*]. β –coefficient represents the change in log relative risk per unit increase in the relevant covariate. Each model includes each covariate as an explanatory variable and the log relative risk as the outcome variable.

#### Publication bias

**Figure 5:**
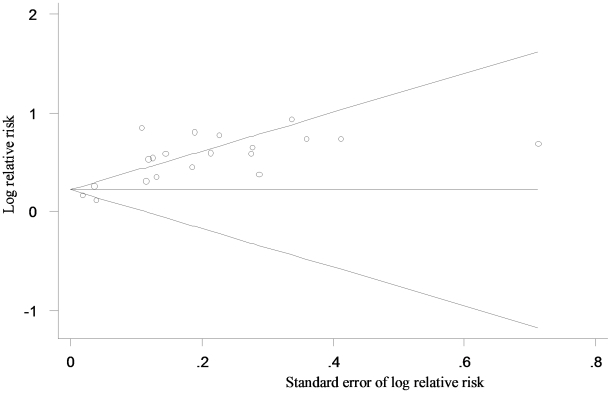
Begg's Funnel Plot* for visual assessment of the presence of publication bias for all studies included in the meta-analysis (each study is represented by an open circle). *Tests for Publication Bias. *For Prospective Cohort Studies (n = 9)*, *Egger's bias 2.50 (p 0.011)*. *For Cross-Sectional Studies (n = 13)*, *Egger's bias 2.64 (p<0.001)*. *Overall Egger's bias p<0.001*.

A funnel plot indicated the presence of publication bias in these studies (Figure 5). Significant publication bias was also observed using Egger's bias test (p = 0.011 for prospective cohort and p<0.0001 for cross-sectional studies).

#### Confounding

Since limited information was available from the published studies, we were unable to assess the impact of confounding based on results from published studies. Instead, we assessed the impact of confounding in the EPIC-Norfolk cohort as described below.

### B. EPIC-Norfolk cohort

We assessed the impact of confounding in EPIC-Norfolk study participants with detailed covariate information (499 incident cases, 15,051 non-cases). The following were associated with lower WBC counts: female sex, lower BMI, lower waist circumference, lower age, never smoking (Table 2).

**Table 2:** Distribution of T2D risk factors according to tertiles of total WBC count at baseline, EPIC-Norfolk Study.

Total WBC tertiles	1 (n = 5,477)	2 (n = 5,120)	3 (n = 4,953)	*P* for trend
Tertile range, * 10(9)/L	1–5.8	5.8–7.0	7.1–40.5	
**Sociodemographic variables**				
Age, y	57.6±9.3	58.1±9.3	58.0±9.5	0.01
Sex, n (% female)	3,137 (57.3)	2,778 (54.3)	2,677 (54.1)	<0.001
Education level, n (%)				0.004*
‘A’ level† and above	3,045 (55.6)	2,867 (56.0)	2,622 (52.9)	
Below ‘A’ level†	2,432 (44.4)	2,253 (44.0)	2,331 (47.06)	
**Anthropometric measures**				
BMI, Kg/m^2^	25.8±3.6	26.3±3.8	26.5±3.8	<0.001*
Waist circumference, cm	86.3±11.9	88.2±12.2	89.2±12.8	<0.001*
**Health related behaviours**				
Physical activity level, n (%)				<0.001*
Active	1,107 (20.2)	953(18.6))	856 (17.3)	
Moderately active	1,294 (23.6)	1,157(22.6)	1,084(21.9)	
Moderately inactive	1,566(28.6)	1,501 (29.3)	1,346 (27.2)	
Inactive	1,510 (27.6)	1,509 (29.5)	1,667 (33.7)	
Smoking status, n(%)				<0.001*
Never	2,961 (54.1)	2,417 (47.2)	1,933(39.0)	
Former	2,244 (41.0)	2,186 (42.7)	1,939 (39.2)	
Current	272 (5.0)	517 (10.1)	1081 (21.8)	
**Medical history**				
Family history of diabetes present, n (%)	682(12.5)	676 (13.2)	644 (13.0)	0.32*

1 Data are means ± standard deviation. *P* values are derived using the Kruskal-Wallis test for continuous variables and the chi-squared test for categorical variables.

† ‘A’ level  =  Advanced Level General Certificate of Education, ‘O’ level  =  Ordinary Level General Certificate of Education.

*age and sex adjusted.

Table 3 shows hazard ratios for incident T2D by tertiles of total WBC and sub-fractions distribution. We decided *a priori* to include T2D family history, physical activity and educational level in our models since they have been previously associated with peripheral WBC [45]–[48]. The age and sex adjusted HR for WBC count of 2.19 (95% CI: 1.74; 2.75) was reduced to 1.82 (95% CI: 1.45; 2.29) after further accounting for smoking status, T2D family history, physical activity, education, BMI and waist circumference, comparing the top and bottom tertiles of the total WBC distribution. Adjusting only for age, sex, smoking, BMI, waist circumference the HR for WBC count was 1.82 (95% CI 1.44, 2.29), comparing the top and bottom tertiles of the total WBC distribution.

**Table 3:** Hazard Ratio (95% CI) of incident T2D by tertiles of total WBC and sub-fractions.

	Lowest tertile	Middle tertile	Highest tertile	*P* for trend
**WBC (n)**	5,477	5,120	4,953	
Mean **10^3^ cells L^−1^**	4.9	6.4	8.5	
Diabetes (n)	111	160	228	
Range	≤5.7	5.8–7.0	≥7.1	
Model 1	1	1.47 (1.17–1.90)	2.19 (1.74–2.75)	<0.001
Model 2	1	1.33 (1.04–1.70)	1.82 (1.45–2.29)	<0.001
**Granulocytes (n)**	5,499	4,940	5,111	
Mean **10^3^ cells L^−1^**	2.7	3.8	5.5	
Diabetes (n)	131	155	213	
Range	≤3.3	3.4–4.3	≥4.4	
Model 1	1	1.28 (1.01–1.61)	1.68 (1.35–2.08)	<0.001
Model 2	1	1.14 (0.90–1.43)	1.45 (1.17–1.81)	0.001
**Lymphocytes (n)**	5,646	5,319	4,585	
Mean **10^3^ cells L^−1^**	1.44	1.97	2.78	
Diabetes (n)	134	151	214	
Range	≤1.7	1.8–2.2	≥2.3	
Model 1	1	1.21 (0.96–1.53)	2.02 (1.63–2.51)	<0.001
Model 2	1	1.09 (0.86–1.37)	1.66 (1.33–2.06)	<0.001
**Monocytes (n)**	5,754	5,899	3,897	
Mean **10^3^ cells L^−1^**	0.23	0.48	1.00	
Diabetes (n)	156	199	144	
Range	≤0.3	0.4–0.6	≥0.7	
Model 1	1	1.17 (0.94–1.44)	1.22 (0.97–1.54)	0.07
Model 2	1	1.10 (0.90–1.36)	1.14(0.90–1.42)	0.268

Model 1: adjusted for age and sex (n = 15,550).

Model 2: as model 1 plus smoking status, family history of diabetes, physical activity level, education level, BMI and waist circumference (n = 15,550).

#### Analysis within participants with an HbA1c of less than 6.5% at baseline

Baseline HbA1c data were available for only a subset of EPIC-Norfolk (n = 9558). 9392 individuals had a baseline HbA1c of less than 6.5%, 166 of whom developed incident diabetes. The age and sex adjusted HR for WBC count of 2.07 (95% CI: 1.41 3.04) was reduced to 1.87 (95% CI: 1.27; 2.76) after further accounting for smoking status, T2D family history, physical activity, education, BMI and waist circumference, comparing the top and bottom tertiles of the total WBC distribution.

#### Analysis within normal total WBC count limits

When restricting analyses to individuals with WBC counts within the normal range (4 to 11*10^9^/L) results were similar to when including participants irrespective of the normal range (as in Table 3). Comparing the top to bottom tertile of the relevant distribution, the HR was 1.87 (1.47; 2.39) for total WBC count, 1.45 (1.15–1.83) for granulocytes, 1.73 (1.37–2.17) for lymphocytes and 1.11 (0.88–1.41) for monocytes.

#### Analysis with Health Behaviours score (HBS)

Given the associations between WBC and the range of heath behaviours, we investigated whether associations between WBC count and T2D differed according to groups defined by adverse versus healthier lifestyle choices, assuming that unmeasured confounders clustered according to groups defined by measured behaviours. When comparing models including HBS, family history of diabetes, education level, BMI and waist circumference with and without an interaction term between WBC and HBS, there was no significant difference between the models, so no evidence of an interaction was detected (p for interaction 0.21).

## Discussion

### Summary of Findings

The present meta-analysis includes evidence about the association between WBC count and T2D from 20 cross-sectional and prospective cohort observational studies, comprising a total of 8,647 T2D cases and 85,040 non cases. Total WBC count was significantly associated with T2D, after adjustment for age, sex, smoking, BMI, waist circumference. Total granulocyte (and subset neutrophil) as well as lymphocyte but not monocyte count were also significantly associated with T2D, after adjustment for age, sex, smoking, BMI, waist circumference The findings were similar for incident T2D in the EPIC-Norfolk cohort analysis, where further adjustment for measured confounders showed the potential for residual confounding.

The available biological data have strongly suggested that T2D is an inflammatory disease [1]–[5].Various markers of inflammation predict the future diabetes risk, including IL-6, CRP[1], sialic acid, and orosomucoid[5]. An inflammation score that included the above four parameters at baseline increased the future T2D risk almost four fold, when comparing the extreme quintiles in non-smoking individuals[49]. A recent analysis from the ARIC cohort showed that WBC, a marker for inflammation, contributed to the short term increased risk of T2D among participants recently quitting smoking[50].

We did not find an increased risk of incident T2D for participants belonging to the higher monocyte tertile. Our findings agree with the previously reported results of the ARIC study [9]. Several stimuli, including pro-inflammatory as well as metabolic stimuli increase the recruitment of monocytes to peripheral tissues, where they differentiate to macrophages and dendritic cells [51]. The destination of monocytes is therefore not the bloodstream and hence peripheral enumeration is not representative of monocyte tissue presence or a possible local monocyte-mediated tissue effect.

Possible mechanisms that could link inflammation and diabetes include interruptions of the insulin signalling in the liver by inflammatory molecules like IL-6 [52]or a pro-inflammatory effect on insulin[53], or insulin resistance[54], [55]. Obesity, a major risk factor for diabetes, is a state of chronic inflammation and is associated with elevated levels of CRP[56], IL-6 [6] and plasminogen activator inhibitor-1 (PAI-1) [7]. Thus, it is possible that the association between inflammation and T2D is mediated by obesity. Studies involving tight matching for obesity suggest that there is an association between WBC count and insulin resistance but may be subject to residual confounding[47].In the current analyses, we adjusted for both waist circumference as well as BMI to control for confounding associated with obesity. Also, we investigated possible sources of confounding in this report using the EPIC-Norfolk study. The observation that the effect size, adjusted for a wide range of possible confounding factors, was lower that that adjusted for age and sex alone does suggest the potential for confounding. Of course, residual confounding by factors not considered at all or by factors we have considered but measured imprecisely can not be excluded.

### Limitations

Measurement error could affect our assessment of exposures, outcomes and confounding factors. Differences in the methods used to measure WBC counts as well as different performance of the same assays at different time periods might have contributed to error in longitudinal studies. In general, such measurement error would attenuate the measure of association if non-differential with regard to the case status. Some cases of type 1 diabetes (T1D) might have been inadvertently included; however, such misclassification is likely to have a minimal impact on effect sizes, given that T1D constitutes only 5–10% of all diabetes cases. Also, given the ascertainment methods used, misclassification is unlikely in prospective cohorts; all except three (NHEFS-NHANES I, NHANES III, Pima Papago Gila River) included incident cases occurring after age 35 years. The majority of cross-sectional studies asked about the type of diabetes when assessing prevalent diabetes or excluded participants using insulin within the first year of diagnosis.

It is possible that hyperglycaemia itself has an impact on WBC levels. In people with diabetes, WBC levels are lowered by treatment with rosiglitazone[57], [58] which may be due to the lowering of glucose levels or an immunomodulatory effect of this class of drugs. However, similar reductions in WBC have been observed with other types of glucose lowering drugs including acarbose[59]. To investigate the possibility of reverse causality, we compared the difference in strength of the association with T2D between cross-sectional and prospective cohort studies. The higher combined RR of WBC in cross-sectional compared to prospective cohort studies could be suggestive of reverse causality. However, since the difference between combined RR between cross-sectional and cohort studies was not statistically significant and because of evidence of presence of publication bias, reverse causality is less likely. Moreover, limiting analyses to healthier people at baseline by excluding participants with a possible or confirmed history of chronic diseases including T2D at baseline, and limiting the analysis to individuals with a baseline HbA1c of less than 6.5%, the risk estimate was almost identical to that in the entire dataset further decreasing the attractiveness of a reverse causality hypothesis, at least in the EPIC-Norfolk cohort.

Results of this study are based on a systematic and comprehensive literature review, including data from both prospective cohorts and cross-sectional studies, and previously unpublished data for a large number of participants. Incomplete retrieval of available results and studies is possible, since the studies included were all published in the English language. However, additional searches showed that no article in a language other that English that fitted the inclusion criteria could be identified. To address variations in design and diabetes ascertainment we asked corresponding authors to report their cases following the same diabetes definition (WHO 1999) and standardize their effect estimates for the same set of covariates (age, sex, smoking, BMI and waist circumference). Despite these attempts of standardisation, heterogeneity between investigator sought studies was not totally eliminated. The funnel plot does indicate the potential for publication bias despite our efforts to obtain data that were not published. This could have been due to the fact that our search strategy, although exhaustive, was more likely to identify studies with reported results on WBC and T2D associations. Sensitivity analyses revealed a significantly lower RR in larger (≥500 cases) compared to smaller studies but no significant difference comparing published to unpublished evidence.

### Summary of conclusions

In summary, these results suggest that WBC is positively associated with the risk of T2D. However, the presence of publication bias and failure to control for all potential confounders in all studies suggests that the observed association may be an overestimate of the truth. We cannot exclude the possibility of reverse causality or residual confounding. Approaches such as the use of genetic determinants of WBC as instrumental variables may be useful to deal with these as yet unresolved issues.

## Supporting Information

Table S1 Summary of the studies of association between WBC and T2D included in the meta-analysis
(0.09 MB RTF)

PRISMA Checklist S1 PRISMA Checklist of items to include when reporting a systematic review or meta-analysis (diagnostic review consisting of cohort studies)
(0.07 MB DOC)
