# Supplementary material for: Differential White Blood Cell Count and Type 2 Diabetes: Systematic Review and Meta-Analysis of Cross-Sectional and Prospective Studies
Source: PLoS One. 2010 Oct 18;5(10):e13405. doi: 10.1371/journal.pone.0013405 (PMC2956635; doi:10.1371/journal.pone.0013405)
Supplement: Table S1 — Summary of the studies of association between WBC and T2D included in the meta-analysis (0.09 MB RTF) [file pone.0013405.s001.rtf]

Study	Data source	Type /follow up
mean or median	Diabetes Definition	Cases/Non-cases	WBC measurements (coefficient of variation) 	
Pima/ Papago Gila River study	Paper[10]	Longitudinal /5.5 y	Fasting plasma glucose exceeding 7.8mmol/L(140.4 mg.dl)	54/218	NS	
NHEFS-NHANES I	Paper [20]	Longitudinal /20 y	self report, hospital discharges, death certificates	878/7474	Beckman Coulter FN cell counter	
ARIC	Paper [9]	Longitudinal /7y 	Diagnosis by a physician, or use of hypoglycaemic medication, a fasting glucose value of more than 7·0 mmol/L (fasting) or 11.1 mmol/L (random)	1335/10,995	Automated cell counter	
Chin-Shan Community Cardiovascular Study	Paper [21]	Longitudinal /10 y	fasting plasma glucose ≥ 7.0mmol/l or use of diabetic medications	548/2412	Sysmex Cell Counter	
Korean Men and Women	Paper [22]	Cross-sectional	fasting plasma glucose ≥ 7.0mmol (126 mg/dl)	NA/15,654	Sysmex Cell Counter	
NHANES III	Paper [23]	Cross-sectional	WHO 1999  or use of diabetes medications 	750/6597	NHANES III protocol, National Center for Health	
The Western New York Study	Paper [24]	Longitudinal / 6y 	Fasting plasma glucose exceeding 125 mg/dl or use of diabetic medications at follow-up	61/158	Automated cell counter	
The Cardiovascular Health Study	Author data	Longitudinal/ 3.5 y	WHO 1999 or use of antidiabetic medications at third visit	237/1724	automated instruments, at local haematology laboratories near each field centre	
EPIC Norfolk	Author data	Longitudinal /14 y	Multiple sources of ascertainment 	499/15,051	Coulter Corporation Haematology Analyzer (≤3%) 	
I-Lan county Study	Author data	Cross-sectional	WHO 1999	157/912	NS	
CURES	Author data	Cross-sectional	WHO 1999	1170/850	Sysmex Cell Counter	
PIVUS	Author data	Cross-sectional	WHO 1999	89/922	Measured by standard laboratory techniques at the Uppsala University Hospital	
Guangzhou Biobank Cohort Study	Author data	Cross-sectional	WHO 1999	1523/8620	Sysmex Cell Counter	
King Chulalongkorn Memorial Hospital	Author data	Cross-sectional	WHO 1999	92/1286	Sysmex Cell Counter, 3.6%	
The Pianoro Study	Author data	Cross-sectional	WHO 1999	165/965	Bayer Advia Counter	
Workplaces study, Nagoya Japan	Author data	Cross-sectional	WHO 199	249/3345	Automated particle counters	
City of Västerås Study	Author data	Cross-sectional	WHO 1999	78/348	GMI Abbott Cell-Dyn 3500	
RIAD	Author data	Cross-sectional	WHO 1999	72/301	NS	
Taichung Health Screening	Author data	Cross-sectional	WHO 1999	667/6793	Sysmex Cell Counter	
Pune Study	Author data	Cross-sectional	WHO 1999	23/415	NS	

WHO 1999 [11] criteria are: fasting plasma glucose ≥ 7.0mmol/l (126mg/dl) or 2–h plasma glucose ≥ 11.1mmol/l (200mg/dl). y = years. NS: not stated 
